# The impact of COVID-19 on nurses’ job satisfaction: a systematic review and meta-analysis

**DOI:** 10.3389/fpubh.2023.1285101

**Published:** 2024-01-11

**Authors:** Yasin M. Yasin, Albara Alomari, Areej Al-Hamad, Vahe Kehyayan

**Affiliations:** ^1^Department of Nursing and Midwifery, Collage of Health Sciences, University of Doha for Science and Technology, Doha, Qatar; ^2^Daphne Cockwell School of Nursing, Faculty of Community Services, Toronto Metropolitan University, Toronto, ON, Canada; ^3^Department of Healthcare Management, College of Business Management, University of Doha for Science and Technology, Doha, Qatar

**Keywords:** job satisfaction, COVID-19, systematic review, healthy work environment, healthcare management

## Abstract

**Background:**

The global healthcare landscape was profoundly impacted by the COVID-19 pandemic placing nurses squarely at the heart of this emergency. This review aimed to identify the factors correlated with nurses’ job satisfaction, the impact of their job satisfaction on both themselves and their patients, and to explore strategies that might have counteracted their job dissatisfaction during the COVID-19 pandemic.

**Methods:**

The Joanna Briggs Institute (JBI) methodology for systematic reviews of prevalence and incidence was used in this review. The electronic databases of CINAHL, MEDLINE, SCOPUS, PsycINFO and Academic Search Complete were searched between January 2020 to February 2023.

**Results:**

The literature review identified 23 studies from 20 countries on nurses’ job satisfaction during the COVID-19 pandemic. A pooled prevalence of 69.6% of nurses were satisfied with personal, environmental, and psychological factors influencing their job satisfaction. Job satisfaction improved psychological wellbeing and quality of life, while dissatisfaction was linked to turnover and mental health issues.

**Conclusion:**

This systematic review elucidates key factors impacting nurses’ job satisfaction during the COVID-19 pandemic, its effects on healthcare provision, and the potential countermeasures for job dissatisfaction. Core influences include working conditions, staff relationships, and career opportunities. High job satisfaction correlates with improved patient care, reduced burnout, and greater staff retention.

**Systematic review registration:**

https://www.crd.york.ac.uk/prospero/display_record.php?ID=CRD42023405947, the review title has been registered in PROSPERO and the registration number is CRD42023405947.

## 1 Introduction

The COVID-19 pandemic significantly affected multiple dimensions of healthcare systems across the globe, with nurses being at the epicenter of this crisis (1, 2). In the midst of the COVID-19 outbreak, nurses encountered distinct stressors encompassing both personal and professional spheres, which have the potential to substantially affect their levels of job satisfaction and intentions to remain in their positions (3). Nurses experiencing anxiety related to COVID-19 exhibited higher levels of work-related stress, greater inclination towards leaving their jobs (4). Furthermore, nurses reported a marked increase in their burnout level (5). As fundamental healthcare professionals accountable for patient care, nurses have grappled with unparalleled challenges that include an escalation in workload (6), scarcity of resources, vulnerability to infection (7), and emotional distress (8). The emotional toll from seeing increased patient morbidity and mortality during the pandemic, and the limited psychological support provided by the organization, significantly affected nurses’ emotional wellbeing and job satisfaction (9). Reduction in salaries and increased workload played a significant role as well, with adequate remuneration and acknowledgment acting as motivational factors (9).

Nurse’s job satisfaction during COVID-19 was a critical factor affecting their performance, productivity, and retention (10). Nurses experiencing low job satisfaction may be prone to burnout (11), reduced job performance (12), and increased likelihood of leaving the profession (10). Furthermore, job dissatisfaction may negatively impact nurses’ mental and physical health, exacerbating stress, anxiety, and other mental health conditions (1, 6, 10). Low job satisfaction among nurses during the COVID-19 pandemic could have had far-reaching consequences, not only for themselves but also for patient care and healthcare organizations as a whole (10).

Recognizing the significance of addressing job satisfaction among nurses, especially during the COVID-19 pandemic, the implementation of effective mitigation strategies is imperative (3, 13). These strategies may include promoting supportive work environments (8), providing adequate resources and training (7, 13), fostering open communication channels (14), and offering mental health support services (8).

Although the repercussions of the COVID-19 pandemic on various facets of healthcare and the welfare of healthcare professionals have been extensively acknowledged (8), there is a notable scarcity of exhaustive scholarly literature specifically zeroing in on nurses’ job satisfaction amidst this unparalleled crisis. Consequently, all-encompassing research, inclusive of systematic reviews scrutinizing the distinct impact of the pandemic on nurses’ job satisfaction, has been sparse. Holistic studies centered on nurses’ job satisfaction during the COVID-19 outbreak can furnish critical insights into the specific factors shaping job satisfaction levels, the ramifications of job discontentment on nurses’ wellbeing and the caliber of patient care, and the formulation of efficacious ameliorative strategies. Such research may guide evidence-based interventions and policies to bolster job satisfaction and foster the resilience of nurses, which in turn may catalyze the enhancement of healthcare delivery during and in the aftermath of the pandemic. The aim of this systematic review is to investigate how the COVID-19 pandemic affected nurses’ job satisfaction. Specifically, the review seeks to identify factors influencing job satisfaction, explore the consequences of job dissatisfaction on both nurses and their patients, and examine mitigation strategies employed to counteract job dissatisfaction during the pandemic.

## 2 Methods

This systematic review was conducted in accordance with the JBI methodology for systematic reviews of prevalence and incidence (15) and the Preferred Reporting Items for Systematic Reviews and Meta-Analysis (PRISMA) guidelines (16). *A priori* protocol was registered in PROSPERO and the registration number is (CRD42023405947).

### 2.1 Search strategy

In March 2023, a three-step search strategy was used aimed at locating published studies in English. First, an initial limited search of MEDLINE and the Cumulative Index to Nursing and Allied Health Literature (CINAHL) was undertaken to identify articles on the topic. Text words contained in the titles and abstracts of relevant articles, and index terms used to describe the articles were used to develop a full search strategy.

In the second step in the search strategy, all identified keywords, index terms and MESH terms were adapted for each included database and/or information source. The following databases were searched: CINAHL, MEDLINE, SCOPUS, PsycINFO, and Academic Search Complete. The Boolean operators AND/OR were used to narrow or broaden the search using a combination of the keywords. Search terms included: (nurse or nurses or nursing) AND (“job satisfaction” or “work satisfaction” or “employee satisfaction”) AND (determinant or factor or cause or influence or influencer or predictor or mitigation or prevention or reduction). Finally, the reference lists of the included studies were searched manually to identify any relevant studies.

### 2.2 Study types and participants

Any primary experimental, quasi-experimental, cohort, or cross-sectional research studies that investigated job satisfaction among nurses during the COVID-19 pandemic were included. Studies published in English and between January 2020 to February 2023 were included as this was the time when COVID-19 was declared as a pandemic across the globe. Studies that investigated job satisfaction among nurses outside the COVID-19 pandemic period were excluded. Qualitative studies were also excluded.

### 2.3 Study selection

**Figure 1:**
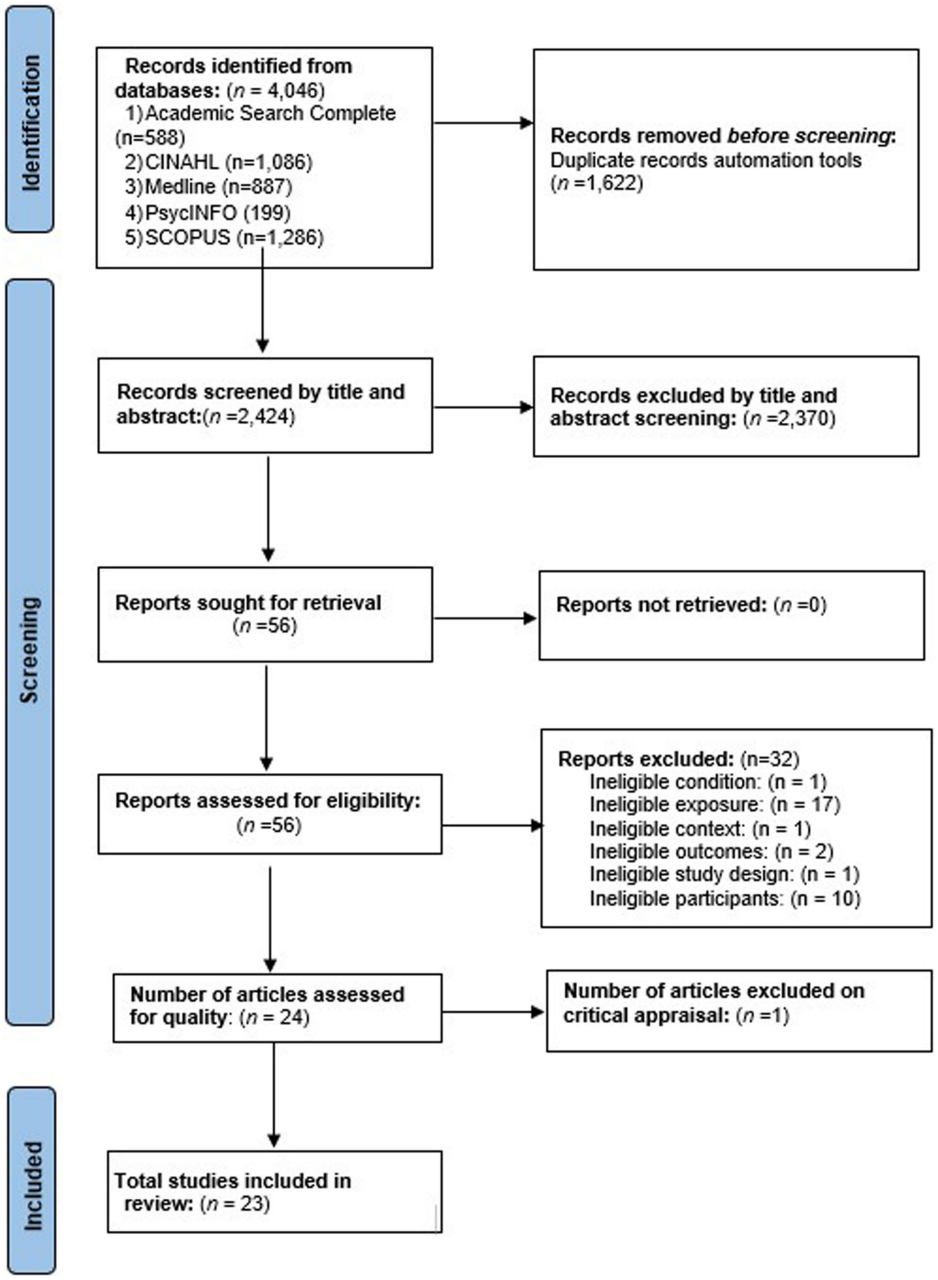
PRISMA flow diagram.

Following the search, all identified citations were collated and uploaded into EndNote version 20 (17) and duplicates were removed. Following a pilot test, titles and abstracts were screened by two independent reviewers (AA, YY) for assessment against the review’s inclusion criteria. Potentially relevant studies were retrieved in full, and their citation details imported into the JBI System for the Unified Management, Assessment and Review of Information (JBI SUMARI). The full text of selected citations was assessed in detail against the inclusion criteria by the two reviewers (AA, YY). Any disagreements that arose between the reviewers at each stage of the selection process was resolved through discussion. The results of the search and the study inclusion process were reported in full in the final systematic review and presented in a Preferred Reporting Items for Systematic Reviews and Meta-analyses (PRISMA) flow diagram (16). See Figure 1.

### 2.4 Assessment of methodological quality

Eligible studies were critically appraised independently by the two reviewers (AA, YY) at the study level for methodological quality using standardized critical appraisal instruments from JBI for experimental (18), quasi-experimental (19), and cross-sectional studies (20). The authors of papers were contacted to request missing or additional data for clarification, where required. Any disagreements that arose were resolved through discussion. The results of the critical appraisal were reported in narrative form and in a table (See Supplementary File 1). Not applicable or unclear answers were considered as not achieved. Any study that received a score of less than 50% on the quality assessment questions was excluded. The Supplementary File 1 shows the detailed methodological quality scores, the questions asked, and the answer key to each study according to the design.

### 2.5 Data extraction

Data extraction from the studies included in the review was carried out independently by the two reviewers (AA, YY) using the standardized data extraction tool for prevalence and incidence available in JBI SUMARI. To resolve extraction discrepancies, a third reviewer was consulted (VK). The data extracted included specific details about the condition, populations, study methods, and proportions of interest to the review specific objectives. For the purpose of meta-analysis, the pooling of estimates was executed by using JBI SUMARI. The transformation of data was done by applying a random-effects model that employed the Freeman–Tukey transformation. In order to assess heterogeneity, the standard *I*^2^ tests were judiciously employed. In situations where statistical pooling was deemed unfeasible, the outcomes were presented through a comprehensive narrative, supplemented with tables and figures in order to aid in the presentation of data.

## 3 Results

The literature search generated a total of 4,046 citations. Among these, 1,622 were identified as duplicates and were subsequently removed. The remaining 2,424 citations underwent a preliminary screening process, where titles and abstracts were examined for relevance according to the inclusion criteria. Based on this screening, 56 citations were selected for a comprehensive assessment involving full-text review. Within this subset of 56, 32 studies were also excluded for various reasons, including: ineligibility based on the condition under investigation (*n* = 1), exposure (*n* = 17), context (*n* = 1), outcomes (*n* = 2), study design (*n* = 1), and participant characteristics (*n* = 10). A critical appraisal was conducted on the remaining 24 studies, during which one study was further excluded due to a quality assessment score below the 50% threshold. Consequently, a total of 23 studies met the inclusion criteria and were incorporated in the final review.

### 3.1 Characteristics of included studies

The final selected studies were carried out from February 2020 to February 2022, during which the primary focus of data collection was in the peak period of the COVID-19 pandemic. Out of these, the majority of the included studies (21 out of 23) utilized a cross-sectional survey methodology. One study was open-label randomized controlled trial (21), and one study utilized a quasi-experimental design (22). The research had taken place across 20 different countries, with two of the studies being multi-national (23, 24). The countries that were part of this review included Bangladesh, Brazil, Canada, China, Egypt, Germany, Great Britain, Hong Kong, Iran, Israel, Italy, Philippines, Poland, Portugal, South Korea, Spain, Sweden, Switzerland, Turkey, and the United States. There was a diverse range in the number of participants in these studies, with the smallest sample consisting of 52 and the largest including 4,561 participants. The aggregate number of participants involved in all the studies exceeded 17,196. The settings in which these studies were conducted varied but mainly included hospital settings, specifically COVID-19 units, emergency departments, tertiary hospitals, isolation wards, inpatient hospital settings, outpatient clinics, and community facilities. Further details are provided in Supplementary File 2.

### 3.2 Level of job satisfaction

**Figure 2:**
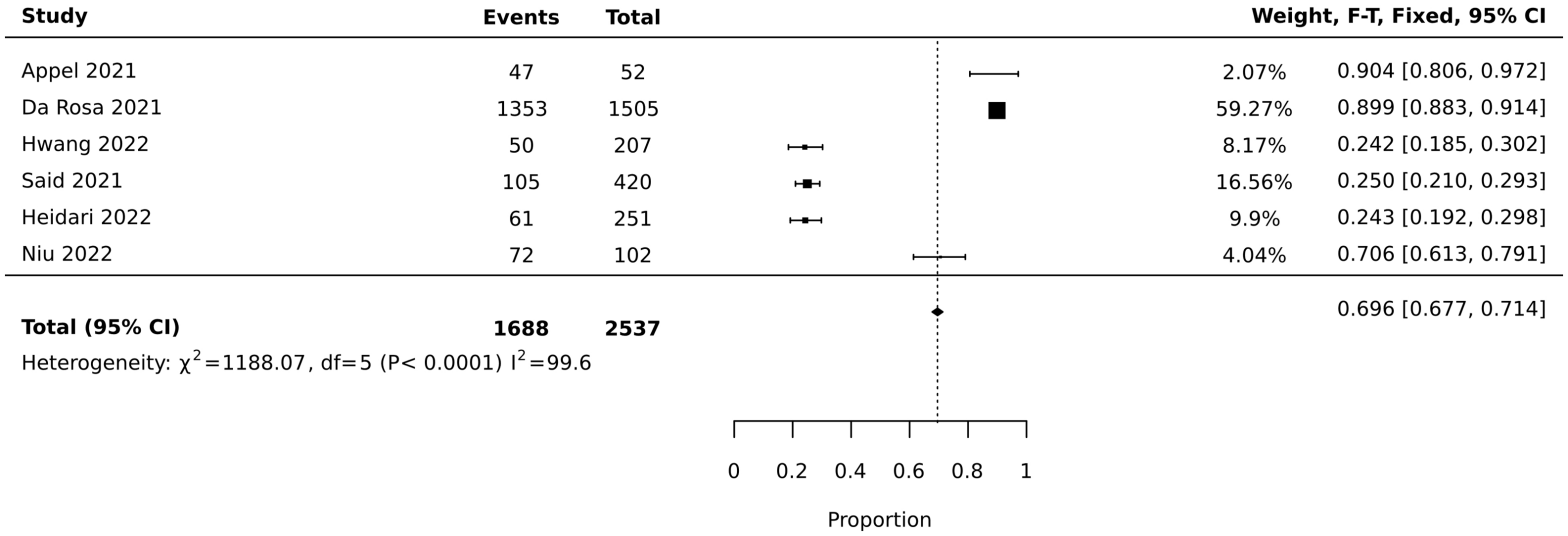
Forest Plot and Meta-analysis of nurses job satisfaction.

Six studies reported the percentage of nurses’ job satisfaction during the pandemic. Of these, two provided specific cutoff points on their respective scales to delineate job satisfaction (25, 26); three presented job satisfaction data in terms of prevalence percentages (27–29); and one study utilized a 10-point single-item scale and applied a median split to categorize nurses’ job satisfaction into high and low (30). The pooled data from these six studies indicated that 69.6% (95% CI: [67.7, 71.4%]) of nurses were satisfied with their jobs, as illustrated in Figure 2. Specifically, three of the studies reported that nurses’ satisfaction level was more than 70% (27, 29, 30). Three other studies reported that their job satisfaction was 24.2 to 25% (25, 26, 28). The remaining studies reported means and standard deviations without categorical description of job satisfaction levels. See Supplementary File 2.

### 3.3 Tools used to assess job satisfaction

The 23 included studies employed a diverse set of measurement tools to evaluate job satisfaction. Among these tools were the McCloskey/Mueller Satisfaction Scale (23, 26), the Minnesota Satisfaction Questionnaire (25), the Turkish Job Satisfaction Scale (31), the Job Satisfaction Scale (24), the Safety Attitudes Questionnaire (32), the Nursing Questionnaire on Organizational Health (22), and the Brayfield and Rothe’s 5-item Short Index of Job Satisfaction (33). Some of the researchers though opted for a single item scale to gauge job satisfaction (27, 29, 30, 34–38). Other researchers adapted subscales of existing questionnaires; for instance, four questions focusing on work satisfaction were adapted from Shaver and Lacey (39). The job satisfaction subscale of the UNIPSICO Battery was employed in a similar manner (14). Sampaio et al. used the job satisfaction dimension from the Copenhagen Psychosocial Questionnaire’s (40), while Goktas et al. employed a 5-point scale that was a refined version of Brayfield and Rothe’s scale (21). Savitsky et al. constituted an occupational satisfaction scale by utilizing items from the Minnesota Satisfaction Questionnaire, the Measure of Job Satisfaction, and other items (41). Finally, Barili et al. constructed an index “Satisfaction i” to measure job satisfaction derived from items extracted from the Labor Force Survey (42).

### 3.4 Factors affecting job satisfaction

The review concluded that several factors significantly contributed to better job satisfaction in nurses. These statistically significant factors were categorized into personal and demographic factors, work environment factors, and psychological and emotional factors.

#### 3.4.1 Personal and demographic factors

Experience (37), lower education (33), and having a family (33, 36, 37, 42) were personal factors contributing to job satisfaction. Older age was generally associated with greater job satisfaction (32, 36, 37, 42), with an exception in one study (24). One study found that female nurses were less satisfied compared to male nurses (24). Financial matters, particularly salary and earnings satisfaction, were also important (33, 39, 42). Good health positively impacted job satisfaction (42), but COVID-19 infection, especially if led to hospitalization, had a negative impact (31, 42).

#### 3.4.2 Work environment factors

The quality of the work environment, including supportive supervision (39), availability of resources (14, 33, 36), manageable workloads (14, 39), adequate staffing (32, 42), employer and coworker support (32), working in a community compared to hospital setting (41), and effective COVID-19 measures (14, 32, 36, 37, 42) were vital for job satisfaction. Conversely, working in a COVID-19 unit (30, 36, 38, 40, 43), job insecurity (14), or working overtime (42) negatively affected job satisfaction. Employment in government hospitals, training against workplace violence, job performance rewards (33), the geographical location of the health care facility, and perceived job importance (24) also contributed to nurses’ job satisfaction.

#### 3.4.3 Psychological and emotional factors

Changes in work conditions, prestige, and commitment to nursing during COVID-19 negatively impacted job satisfaction (24). Anxiety (36), role conflicts, psychosomatic problems (14), and fear of COVID-19 infection (35) also contributed to decreased job satisfaction. Concerns regarding potential infection and stigma from working in high-risk areas like COVID-19 treatment centers further decreased job satisfaction (36). In contrast, positive behaviors, specifically discretionary efforts aimed at enhanced care for COVID-19 patients, and adaptability post-trauma led to post-traumatic growth and job satisfaction, had favorable effects.” (36, 39).

### 3.5 Consequences of job satisfaction

#### 3.5.1 Positive consequences

Chong et al. shed light on the enhancement of psychological flexibility and mental wellbeing as positive outcomes of job satisfaction (23). Additionally, correlation was reported between better professional quality of life and higher job satisfaction (28, 29). Further, elevated job satisfaction was associated with improved quality of life and wellbeing in the workplace (40). Moreover, higher levels of job satisfaction had a positive impact on nurses’ organizational commitment and their inclination to provide care to COVID-19 patients (39).

#### 3.5.2 Negative consequences

Conversely, job dissatisfaction was associated with several undesired consequences. For instance, job dissatisfaction during COVID-19 was found to be associated with higher turnover intentions (38). Job dissatisfaction was correlated with mental health issues (23). Depression and stress were specifically identified as detrimental consequences of low job satisfaction (27, 30, 34). Anxiety was reported to be negatively affected by low job satisfaction (27, 34, 37). Chowdhury et al. showed that job dissatisfaction resulted in increased workplace violence, bullying, and burnout (33). Moreover, Heidari et al. (25) expounded on burnout as a result of job dissatisfaction, specifically highlighting that emotional exhaustion and compromised personal accomplishment, as facets of burnout, were impacted.

### 3.6 Interventions to enhance job satisfaction during COVID-19

This review identified two studies that investigated the efficacy of interventions aimed at bolstering job satisfaction. Goktas et al. employed a randomized controlled experiment to assess the impact of disseminating motivational messages on the job satisfaction of emergency nurses amidst the COVID-19 pandemic (21). The experimental intervention entailed transmitting motivational text messages to participants in the intervention group three times daily. These messages were crafted to augment job satisfaction and communication skills while mitigating compassion fatigue. The findings of this study evinced a favorable effect of the intervention on the participants’ job satisfaction.

Similarly, Zaghini et al. utilized a longitudinal mixed-methods design to scrutinize the implementation of organizational proactive management interventions and their impact on nurses’ job satisfaction (22). The study gauged job satisfaction before and after the implementation of a spectrum of interventions. These interventions encompassed measures pertaining to the nursing work environment, staffing and workload adjustments, the enhancement of competence and learning, fostering a participatory approach and autonomy, unit-level strategies concerning COVID-19, and surveillance of healthcare nurses. The study ascertained that the execution of these organizational proactive management interventions positively influenced job satisfaction.

## 4 Discussion

The COVID-19 pandemic had a significant impact on all aspects of healthcare systems globally, including healthcare professionals who were in the front lines caring for patients with COVID-19 infections. Of particular concern, which is the subject of this systematic review, is the wellbeing of nurses who experienced serious stressors that had the potential to substantially affect their levels of job satisfaction and commitment to remain in their profession. While considerable number of research studies have been conducted on nurses’ job satisfaction during the COVID-19 pandemic, to our best knowledge, no studies had explored this subject systematically. In planning this systematic review, we believed that our review would provide critical insights into the specific factors during the pandemic that influenced nurses’ job satisfaction, and the impact of job dissatisfaction on their wellbeing and the consequential quality of patient care provided.

### 4.1 Level and measurement of job satisfaction

The results from the meta-analysis indicated a bifurcation in the levels of nurses’ job satisfaction during the pandemic. While the pooled data from six studies showed that approximately 69.6% of nurses were satisfied with their jobs during the pandemic, there was a noticeable divergence between the studies. Half of these studies reported job satisfaction levels exceeding 70% (27, 29, 30), while the other half reported significantly lower levels between 24.2 to 25% (25, 26, 28). One possible explanation for this disparity could be the setting and context in which the studies were conducted. National policies regarding healthcare workers’ remuneration, workload, and support during the pandemic might have been different between different countries (43). The studies reporting higher levels of job satisfaction were primarily conducted in the USA, Brazil, and China. These nations, despite their economic and cultural differences, may have features in their healthcare systems that contribute positively to job satisfaction during the pandemic. In contrast, studies from Iran and Egypt reported lower satisfaction scores, potentially linked to economic challenges, healthcare resource limitations, and cultural perceptions of nursing. Notably, the job satisfaction scores from South Korea presented an unexpected outcome, with levels significantly lower than other countries. This anomaly could be reflective of the particularly high rates of burnout and workload experienced by healthcare providers in South Korea during the pandemic (44). Such stressful working conditions are likely to adversely affect job satisfaction and may have accounted for the distinct results observed in this context. As well, it is important to consider the cultural differences in the way people perceived the subjective questions about job satisfaction when comparing data across countries (45). To comprehensively understand the nuances of job satisfaction among healthcare professionals in these countries, further research is warranted. Such studies should delve into the interplay of economic, cultural, and systemic factors that contribute to these differences, considering the unique resilience measures and support systems for nurses that these nations may have implemented during the pandemic.

### 4.2 Factors affecting job satisfaction

The findings of this review indicated that job satisfaction among nurses was influenced by a combination of personal and demographic factors, work environment factors, and psychological and emotional factors. This is consistent with previous literature which has also shown the multidimensional nature of job satisfaction (46, 47).

Regarding personal and demographic factors, our review found that higher working experience (37), lower education preparation (33), and having a family (33, 36, 37, 42) were linked to higher job satisfaction. These findings may be attributed to the potential stability and familiarity that experience brings (48), and the support systems that families could provide (49). Moreover, this interpretation may explain why older age was generally associated with higher job satisfaction. Kovner et al. suggested that older nurses might have more realistic expectations and coping skills (50). However, Makowicz et al. found an exception regarding age, which suggested that other factors might have moderated the relationship between age and job satisfaction, which might require further studies (24).

Work environment factors were found to be vital in determining job satisfaction. Positive factors included supportive supervision, availability of resources, reasonable workloads, and effective COVID-19 measures (14, 32, 33, 36, 37, 39, 42). Our findings are consistent with the conclusions of Persefoni’s review (51), which synthesized evidence of a significant association between nurses’ job satisfaction and the quality of their work environment. Interestingly, nurses working in community settings were found to have higher job satisfaction than those in hospital settings (41), possibly due to lower patient acuity and more autonomous practice.

Conversely, psychological and emotional factors such as anxiety, role conflicts, and fear of COVID-19 infection contributed to reduced job satisfaction (14, 35, 36). This supports the findings of Lee and Jang (52), who identified that emotional status had a significant impact on nurses’ job satisfaction. Concerns regarding potential infection and stigma from working in high-risk areas like COVID-19 treatment centers further decreased job satisfaction (36), underscoring the significant impact of the pandemic on nurses’ psychological wellbeing and job satisfaction.

### 4.3 Consequences of job satisfaction

The consequences of job satisfaction among nurses, especially during the COVID-19 pandemic, are multifaceted and crucial to understand for the betterment of healthcare services. Chong et al. illustrated that higher job satisfaction was associated with an enhancement in psychological flexibility and mental wellbeing (23). This finding is critical for healthcare service quality, aligning with earlier research that underscored the role of mental wellbeing in healthcare professionals in determining patient care quality (53). Moreover, improved professional quality of life resulting from high job satisfaction (28, 29) reiterates the importance of maintaining a positive working environment for better service delivery. Sampaio et al. supported this by associating high job satisfaction with improved quality of life and workplace wellbeing (40). Notably, Sharif Nia et al. established that job satisfaction positively influenced nurses’ commitment to the organization and their willingness to provide care to COVID-19 patients (39). This is particularly significant in the context of a pandemic, as the commitment and dedication of healthcare professionals are paramount in handling healthcare crises (54).

On the contrary, job dissatisfaction has been linked to a plethora of negative consequences. Consistent with earlier research (55), Lavoie-Tremblay et al. found that job dissatisfaction during COVID-19 was associated with higher turnover intentions (38), which can be detrimental to healthcare systems that were already strained by the pandemic. Moreover, Chong et al. correlated job dissatisfaction with mental health issues (23), which is alarming considering the stress and emotional turmoil healthcare professionals already face in pandemic settings (56). Specific mental health issues like depression, anxiety burnout, and stress were also observed as consequences of low job satisfaction (25, 27, 30, 34, 37). Furthermore, the work of Chowdhury et al. showing an association between job dissatisfaction and increased workplace violence, bullying, and burnout (33) is particularly concerning.

The findings in this review elucidate the significant impact job satisfaction has on nurses’ mental wellbeing, professional quality of life, and dedication to care, especially during a health crisis like COVID-19. The negative consequences of job dissatisfaction, including mental health issues, turnover intentions, and burnout, are detrimental not only to healthcare professionals but also to the quality of healthcare delivery. As such, it is imperative that measures be implemented to improve job satisfaction among nurses, thereby positively influencing their wellbeing and the overall effectiveness of healthcare systems if future pandemics or health crises.

### 4.4 Interventions to enhance job satisfaction during pandemics

Regarding interventions aimed at enhancing job satisfaction during the COVID-19 pandemic, this systematic review identified two studies with promising outcomes. Goktas, Gezginci et al. used a randomized controlled experiment focusing on the use of motivational messages to improve job satisfaction among emergency nurses (21). This aligns with other research highlighting the positive impact of motivational messages in nurses’ life satisfaction (57). Another noteworthy study included in the review used a longitudinal mixed methods design to investigate the effects of organizational proactive management interventions (22). This is particularly relevant as several studies have stressed the importance of organizational support in improving nurse’ job satisfaction (58, 59).

Taken together, these findings underscore the importance of multifaceted approaches in enhancing job satisfaction among healthcare professionals during a health crisis such as the COVID-19 pandemic. Motivational support and comprehensive organizational interventions can be critical components in addressing the unique challenges faced by nurses and ensuring their mental wellbeing and satisfaction, which in turn can contribute to better patient care.

### 4.5 Implications

The results of this systematic review have several implications for management, policy, practice, education, and future research. Managers and policymakers should consider implementing interventions such as motivational messaging and organizational proactive management interventions, as identified in the review, to improve job satisfaction among nurses, especially during health crises. Implementing policies that address nurses’ work environment ensuring adequate staffing, and providing support and resources may lead to enhanced job satisfaction. In terms of practice, the emphasis should be on establishing supportive supervision, reasonable workloads, and effective health measures to create a conducive work environment. For education, training programs should focus on equipping nurses with the necessary skills to handle the psychological and emotional challenges of their profession, particularly during a pandemic. Educators should also aim at building resilience in nurses and teaching coping strategies. Furthermore, there is a need for standardized tools for assessing job satisfaction, as the review highlighted the use of diverse measurement tools. Future research should focus on understanding the long-term impact of pandemics on job satisfaction and mental health among healthcare professionals. It is essential to develop evidence-based interventions that may be effectively integrated into the healthcare system to bolster job satisfaction, which in turn, could lead to better patient care and wellbeing among healthcare professionals.

### 4.6 Review limitation

A potential limitation of this review was the exclusive consideration of English-language studies. The utilization of differing methods to measure job satisfaction may have also influenced the aggregation of all the investigations, complicating the production of a holistic conclusion. It is also important to note that despite the diligent search for relevant studies to include in this review, the systematic search process may not have captured all applicable research, leading to possible omissions.

## 5 Conclusion

In conclusion, job satisfaction among nurses is multifactorial and requires an integrated approach to address personal, workplace, and psychological dimensions. During the COVID-19 pandemic, it is especially crucial to ensure that nurses have adequate support and resources to maintain their job satisfaction and wellbeing. This review yielded three principal outcomes: identification of the elements that correlated with nurses’ job satisfaction; analysis of the impact of nurses’ job satisfaction on nurses themselves and their patients; and the exploration of counteractive strategies linked to job satisfaction among nurses during COVID-19. The review meticulously analyzed a range of factors, including working conditions, staff relationships, compensation, and career development opportunities, that are correlated with job satisfaction. It also critically assessed the consequences of job satisfaction levels on both nurses and patients, highlighting the linkages between high job satisfaction and inclination to provide nursing care, reduced nurse burnout, and increased retention rates among nursing staff. Additionally, in recognition of the unique challenges faced during the COVID-19 pandemic, the review investigated various strategies such as organizational support, mental health resources, enhanced communication, and adaptive work environments to mitigate job dissatisfaction among nurses.

## Data availability statement

The original contributions presented in the study are included in the article/Supplementary Material, further inquiries can be directed to the corresponding author.

## Author contributions

YY: Conceptualization, Formal analysis, Investigation, Methodology, Project administration, Software, Supervision, Writing – original draft. AA: Conceptualization, Formal analysis, Methodology, Software, Writing – original draft, Writing – review & editing. AA-H: Conceptualization, Data curation, Formal analysis, Investigation, Methodology, Writing – original draft, Writing – review & editing. VK: Conceptualization, Validation, Writing – review & editing.
