# Supplementary material for: The impact of COVID-19 on nurses’ job satisfaction: a systematic review and meta-analysis
Source: Front Public Health. 2024 Jan 11;11:1285101. doi: 10.3389/fpubh.2023.1285101 (PMC10808441; doi:10.3389/fpubh.2023.1285101)
Supplement: Supplementary file 1 [file Table_1.DOCX]

### **Supplementary file 1: Critical Appraisal Results**

#### The critical evaluation score was determined by taking the ratio of the total criteria met to the total criteria established. Qualified studies underwent individual critical assessment at the study level to gauge methodological rigor, employing standardized evaluation tools from JBI. These were specifically intended for experimental (Barker et al., 2023), quasi-experimental (Tufanaru et al., 2020), and cross-sectional studies (Moola et al., 2020).

#### **Supplementary table 1-A:**

#### Critical Appraisal of Eligible Analytical Cross-Sectional Study

| **Citation** | **Q1** | **Q2** | **Q3** | **Q4** | **Q5** | **Q6** | **Q7** | **Q8** | **Score** |
| --- | --- | --- | --- | --- | --- | --- | --- | --- | --- |
| Appel AP, Carvalho A, Santos RPD. 2021. | Y | Y | U | Y | N | N | Y | Y | 63% |
| Barili E, Bertoli P, Grembi V, Rattini V. 2022. | U | Y | U | Y | Y | Y | U | Y | 63% |
| Chong YY, Frey E, Chien WT, Cheng HY, Gloster AT. 2023. | Y | Y | Y | Y | Y | Y | Y | Y | 100% |
| Chowdhury SR, Kabir H, Mazumder S, Akter N, Chowdhury MR, Hossain A. 2022. | Y | Y | Y | Y | Y | Y | Y | Y | 100% |
| Da Rosa P, Brown R, Pravecek B, Carotta C, Garcia AS, Carson P, et al. 2021. | Y | N | Y | Y | Y | Y | Y | Y | 88% |
| Gimenez-Espert MDC, Prado-Gasco V, Soto-Rubio A. 2020. | Y | Y | Y | Y | U | N | Y | Y | 75% |
| Gorini A, Giuliani M, Fiabane E, Bonomi A, Gabanelli P, Pierobon A, et al. 2022. | Y | Y | Y | Y | Y | Y | U | Y | 88% |
| Hwang E. 2022. | Y | Y | U | Y | U | U | U | Y | 50% |
| Heidari S, Parizad N, Goli R, Mam-Qaderi M, Hassanpour A. 2022. | Y | Y | Y | Y | N | N | Y | Y | 75% |
| Işıklı AG, Şen H, Soydaş D. 2021. | N | N | Y | Y | N | N | Y | Y | 50% |
| Labrague LJ, Santos JAA. 2021. | Y | Y | Y | Y | Y | Y | Y | Y | 100% |
| Lavoie-Tremblay, M., Gélinas, C., Aubé, T., Tchouaket, E., Tremblay, D., Gagnon, M. P., & Côté, J. (2022) | Y | Y | Y | Y | Y | Y | Y | Y | 100% |
| Makowicz D, Lisowicz K, Bryniarski K, Dziubaszewska R, Makowicz N, Dobrowolska B. 2022. | Y | N | Y | Y | N | N | Y | Y | 63% |
| Malinowska-Lipie´n I, Wadas T, Gabry´s T, Kózka M, Gniadek A, Brzostek T, et al. 2022. | Y | N | Y | Y | U | U | Y | Y | 63% |
| Niu A, Li P, Duan P, Ding L, Xu S, Yang Y, et al. 2022. | Y | Y | U | Y | Y | Y | Y | Y | 88% |
| Piotrowski A, Sygit-Kowalkowska E, Boe O, Rawat S. 2022. | Y | U | Y | Y | Y | Y | Y | Y | 88% |
| Said RM, El-Shafei DA. 2021. | Y | Y | Y | Y | Y | Y | Y | Y | 100% |
| Sampaio F, Salgado R, Antonini M, Delmas P, Oulevey Bachmann A, Gilles I, et al. 2022. | Y | Y | Y | Y | N | N | Y | Y | 75% |
| Savitsky, B., Radomislensky, I., & Hendel, T. 2021. | N | N | Y | Y | Y | Y | Y | Y | 75% |
| Sharif Nia H, Arslan G, Naghavi N, Sivarajan Froelicher E, Kaveh O, Pahlevan Sharif S, et al. 2021. | Y | N | Y | Y | N | N | Y | Y | 63% |
| Yeung NCY, Wong ELY, Cheung AWL, Leung CSY, Yeoh EK, Wong SYS. 2022. | Y | Y | Y | Y | Y | Y | Y | Y | 100% |
| Yeung NCY, Wong ELY, Cheung AWL, Yeoh EK, Wong SYS. 2021. | Y | Y | Y | Y | Y | Y | Y | Y | 100% |

#### **Y= Yes, N= No, U = Not applicable or unclear answers**

#### **Supplementary table 1-B**

#### Critical Appraisal of Eligible Randomized Controlled Trial

| **Citation** | **Q1** | **Q2** | **Q3** | **Q4** | **Q5** | **Q6** | **Q7** | **Q8** | **Q9** | **Q10** | **Q11** | **Q12** | **Q13** | **Score** |
| --- | --- | --- | --- | --- | --- | --- | --- | --- | --- | --- | --- | --- | --- | --- |
| Goktas S, Gezginci E, Kartal H. 2022. | Y | N | Y | N | N | N | Y | N | Y | Y | Y | Y | Y | 62% |

#### **Y= Yes, N= No, U = Not applicable or unclear answers**

#### **Supplementary table 1-C**

#### Critical Appraisal of Eligible Quasi-Experimental Study

| **Citation** | **Q1** | **Q2** | **Q3** | **Q4** | **Q5** | **Q6** | **Q7** | **Q8** | **Q9** | **Score** |
| --- | --- | --- | --- | --- | --- | --- | --- | --- | --- | --- |
| Zaghini F, Fiorini J, Livigni L, Carrabs G, Sili A. 2021. | Y | Y | Y | N | N | Y | Y | Y | Y | 78% |

#### **Y= Yes, N= No, U = Not applicable or unclear answers**
