# Supplementary material for: The impact of COVID-19 on nurses’ job satisfaction: a systematic review and meta-analysis
Source: Front Public Health. 2024 Jan 11;11:1285101. doi: 10.3389/fpubh.2023.1285101 (PMC10808441; doi:10.3389/fpubh.2023.1285101)
Supplement: Supplementary file 2 [file Table_2.DOCX]

**Supplementary file 2:** Extraction table of the included studies

| **Study** | **Country & sample** | **Setting/ /Period** | **Research design** | **JS Data collection tool** | **Main description of results** |
| --- | --- | --- | --- | --- | --- |
| Appel, A. P; Carvalho, Ards; Santos, R. P. D. 2021 | Brazil  (*n=*52) | COVID-19 unit at public University Hospital/ May to July 2020. | A cross-sectional survey | A single question with a scale from 0 to 10 | - JS level: 90.5% reported high - Impact of JS (+): NR - Impact of JS (-): Depression and stress - Factors affecting JS (+): NR - Factors affecting JS (-): NR |
| Barili, E; Bertoli, P.; Grembi, V.; Rattini, V. 2022 | Italy  *(n _nurses_=*4,561) | Italian healthcare facilities/ June 15 to August 31, 2020 | A cross-sectional survey | “Satisfaction _i_” constructed using  the questions from the Labor Force Survey | - JS level: NR - Impact of JS (+): NR - Impact of JS (-): NR - Factors affecting JS (+): Age ≥ 60 years, good health status, working in high-quality facility, high salary, and exposed to COVID-19. - Factors affecting JS (-): Dirty team, lack of manpower, less effective response, overtime |
| Chong YY, Frey E, Chien WT, Cheng HY, Gloster AT. 2023. | Switzerland  (*n* = 294)  and  Hong Kong  (*n* = 158) | Inpatient hospital settings, outpatient clinics, and community isolation facilities / January to March, 2021 | A cross-sectional survey | The McCloskey/Mueller Satisfaction Scale | - JS level: NR - Impact of JS (+): Psychological flexibility, mental well-being - Impact of JS (-): Mental health problems - Factors affecting JS (+): NR - Factors affecting JS (-): NR |
| Chowdhury SR, Kabir H, Mazumder S, Akter N, Chowdhury MR, Hossain A. 2022. | Bangladesh (*n*=1,264) | Eight tertiary hospitals/ February 25 to July 10, 2021 | A cross-sectional survey | Brayfield and Rothe’s 5-item Short Index of Job Satisfaction (SIJS-5) | - JS level: Mean (SD)=17.3(3.95), possible score is 5- 25 - Impact of JS (+): NR - Impact of JS (-): Depression, workplace violence, bullying, and burnout. - Factors affecting JS (+): Higher salary, timely pay of salary, working in government health care facilities, married, sufficient equipment, rewards for job performance, training to tackle workplace violence, lower educational level - Factors affecting JS (-): NR |
| Da Rosa P, Brown R, Pravecek B, Carotta C, Garcia A S, Carson P, Callies, D, Vukovich M. 2021 | USA  (*n*=1,505) | Acute care setting, primary care, long term care, veteran’s affair and others/ July 13 to August 13, 2020 | A cross-sectional survey | A single question with a scale from five-point Likert scale | - JS level: 89.9% reported Satisfied/very satisfied - Impact of JS (+): NR - Impact of JS (-): Emotional distress including depression, anxiety and stress - Factors affecting JS (+):NR - Factors affecting JS (-):NR |
| Gimenez-Espert MDC, Prado-Gasco V, Soto-Rubio A. 2020. | Spain  (*n*=92) | Two public hospitals in the Valencian Community (Spain)/ March 29 to April 8, 2020 | A cross-sectional study | Job satisfaction subscale of the UNIPSICO Battery | - JS level: Mean (SD)=2.405(0.80), possible score is 0-4 - Impact of JS (+): NR - Impact of JS (-): NR - Factors affecting JS (+): Information available to nurses, COVID-19 measures, availability of resources - Factors affecting JS (-): Role conflict, workload, job insecurity, psychosomatic problems |
| Goktas S, Gezginci E, Kartal H. 2022. | Turkey  (*n*=60) | Emergency departments / July31, 2021, and August31, 2021. | Open label randomized controlled trial | 5-point scale refined by Judge et al. in 1998 from Brayfield and Rothe's 1951 job satisfaction measure | - JS level: Post intervention Mean (SD)=   - Intervention group= 3.54(0.65)   - Control group= 2.82(0.81) - Impact of JS (+): NR - Impact of JS (-): NR - Factors affecting JS (+): Motivational message - Factors affecting JS (-): NR |
| Gorini A, Giuliani M, Fiabane E, Bonomi A, Gabanelli P, Pierobon A, et al. 2022. | Italy  *(n _nurses_=*176) | Italian hospitals and healthcare institutions/ March 1 till July 16, 2021 | A cross-sectional survey | A single question with were the participants asked to declare if they perceived job satisfaction during the last two weeks. | - JS level: NR - Impact of JS (+): NR - Impact of JS (-): Depression, Anxiety - Factors affecting JS (+): NR - Factors affecting JS (-): NR |
| Heidari S, Parizad N, Goli R, Mam-Qaderi M, Hassanpour A. 2022. | Iran  (*n*=251) | Three hospitals/ August to 11 October 2020. | A cross-sectional survey | Minnesota Satisfaction Questionnaire | - JS level: Low (75.7%) - Impact of JS (+): NR - Impact of JS (-): Emotional exhaustion, Personal accomplishment - Factors affecting JS (+): NR - Factors affecting JS (-): NR |
| Hwang, E. 2022 | Korea  *(n*=207) | Tertiary general hospitals/January 17, and February 5, 2022. | A cross-sectional survey | A single question with three categories: good, moderate, and bad | - JS level: Good=50 (24.2%), Moderate =98 (47.3%), and Bad= 59(28.5%) - Impact of JS (+): Quality of work life - Impact of JS (-): NR - Factors affecting JS (+):NR - Factors affecting JS (-):NR |
| Işıklı AG, Şen H, Soydaş D. 2021. | Turkey  (*n*=132) | A University hospital/ March 2021 and April 2021. | A cross-sectional survey | Turkish Job Satisfaction Scale | - JS level: Mean (SD)=66.19 (12.81), possible score is 20-100 - Impact of JS (+): NR - Impact of JS (-): NR - Factors affecting JS (+): NR - Factors affecting JS (-): Being infected with COVID-19 and hospitalized rather than having outpatient treatment. |
| Labrague LJ, Santos JAA. 2021. | Philippines  (*n*=261) | Five COVID-19 referred hospitals/ No information about the data collection date. | A cross-sectional study | Single question categorizing job satisfaction into four categories | - JS level: Mean (SD)=3.65 (0.99), possible score is 1-5 - Impact of JS (+): NR - Impact of JS (-): NR - Factors affecting JS (+): NR - Factors affecting JS (-): Fear of COVID-19 |
| Lavoie-Tremblay, M., Gélinas, C., Aubé, T., Tchouaket, E., Tremblay, D., Gagnon, M. P., & Côté, J. (2022) | Canada  (*n*=1,705) | Acute care, long-term community care, public health and rehabilitation settings, / July 22 to November 16, 2020 | A cross-sectional study | Single question on a 7-level scale | - JS level: Mean (SD)= 4.16 (1.64), possible score is 1-7 - Impact of JS (+): NR - Impact of JS (-): Turnover intention - Factors affecting JS (+): NR - Factors affecting JS (-): Working with COVID-19 patients |
| Makowicz D, Lisowicz K, Bryniarski K, Dziubaszewska R, Makowicz N, Dobrowolska B. 2022. | Poland, Germany, Italy, Great Britain and Sweden  (*n*=1,012) | The study recruited nurses worked or are working in the ward where patients infected with COVID-19/ January to March 2022 | A cross-sectional survey | Job Satisfaction Scale (SSP) | - JS level: NR - Impact of JS (+): NR - Impact of JS (-): NR - Factors affecting JS (+): Felling of job importance, Country of work (Italy has the highest decrease, followed by UK, Germany, Poland, and lastly Sweden) - Factors affecting JS (-): Gender (female), older age, low manpower, change of work condition perception, change in social status(prestige), change in willingness to continue in nursing |
| Malinowska-Lipie´n I, Wadas T, Gabry´s T, Kózka M, Gniadek A, Brzostek T, et al. 2022. | Poland  (*n*=577) | Hospitals with isolation wards and others with non-infectious diseases wards/ 20 December 2020 to 28 February 2021. | A cross-sectional study | Safety Attitudes Questionnaire (SAQ) | - JS level: Mean (SD)= 66.18 (21.88), possible score is 1-5 - Impact of JS (+): NR - Impact of JS (-): NR - Factors affecting JS (+): Older age, training on COVID-19 treatment, availability of resources, psychological counselling, ability to perform COVID-19 test in the workplace, employer’s and co-workers’ support - Factors affecting JS (-): Insufficient nursing staff |
| Niu A, Li P, Duan P, Ding L, Xu S, Yang Y, et al. 2022. | China  (*n*=102) | COVID-19 front line nurses working in three hospitals / March 15 to 21, 2020 | A cross-sectional study | A single question with four points Likert scale | - JS level: 70.6% were satisfied - Impact of JS (+): Professional quality of life - Impact of JS (-): NR - Factors affecting JS (+): NR - Factors affecting JS (-): NR |
| Said RM, El-Shafei DA. 2021. | Egypt  (*n*=420) | COVID-19 Triage hospital *Vs*. neither triage nor isolation hospital /10^th^ to 24^th^ of April 2020. | A comparative cross-sectional survey | The McCloskey/Mueller Satisfaction Scale | - JS level: High (25%), Moderate (28%), Low (46%) - Impact of JS (+): NR - Impact of JS (-): NR - Factors affecting JS (+): NR - Factors affecting JS (-): Working in COVID-19 dedicated hospital |
| Sampaio F, Salgado R, Antonini M, Delmas P, Oulevey Bachmann A, Gilles I, et al. 2022. | Portugal  (*n*=247) | Portuguese territory /November 2021 to January 2022. | A cross-sectional study | Copenhagen Psychosocial Questionnaire- job satisfaction dimension | - JS level: Mean (SD)= 3.07 (0.93), possible score is 1-5 (inverted scale) - Impact of JS (+): Quality of life, workplace wellbeing - Impact of JS (-): NR - Factors affecting JS (+): NR - Factors affecting JS (-): NR |
| Sharif Nia H, Arslan G, Naghavi N, Sivarajan Froelicher E, Kaveh O, Pahlevan Sharif S, et al. 2021. | Iran  (*n*=648) | Hospitals affiliated by Mazandaran University of Medical Sciences/ March 2020 | A cross-sectional survey | Four questions about work satisfaction adapted from Shaver and Lacey with seven-point Likert scores | - JS level: Mean (SD)=16.96 (7.19), possible score is 7-28 - Impact of JS (+): Organisational commitment and intention to care for patients with COVID-19 - Impact of JS (-): NR - Factors affecting JS (+): Quality of supervisor, extra-role behaviours, pay satisfaction - Factors affecting JS (-): Workload |
| Savitsky, B., Radomislensky, I., & Hendel, T. 2021. | Israel  (*n*=130) | Hospitals and community settings /June to September 2020 | A cross-sectional study | Occupational satisfaction tool which includes items from Minnesota Satisfaction Questionnaire, Measure of Job Satisfaction and other items | - JS level: (SD)=3.6 (0.6), possible score is 1-4 - Impact of JS (+): NR - Impact of JS (-): NR - Factors affecting JS (+): working in a community care compared to hospitals - Factors affecting JS (-): Working with COVID-19 patients |
| Yeung NCY, Wong ELY, Cheung AWL, Leung CSY, Yeoh EK, Wong SYS. 2022. | Hong Kong  (*n*=1,510) | Hospitals and community settings/August 8 to September 22, 2020 | A cross-sectional study | A single question with 5-point Likert scale | - JS level: NR - Impact of JS (+): NR - Impact of JS (-): NR - Factors affecting JS (+): Posttraumatic growth , higher age, being married, having children, satisfaction with workplace pandemic control guidelines - Factors affecting JS (-): Anxiety, dirty team, worry about infecting with COVID-19 (family and self), worries about insufficient supplies, perceived stigma |
| Yeung NCY, Wong ELY, Cheung AWL, Yeoh EK, Wong SYS. 2021. | Hong Kong  (*n*=1,510) | Hospitals and community settings/ August 8 to September 22, 2020 | A cross-sectional study | A single question with five points Likert scale | - JS level: NR - Impact of JS (+): NR - Impact of JS (-): Anxiety - Factors affecting JS (+): age, experience, being married, having children, Satisfaction with workplace pandemic control guidelines - Factors affecting JS (-): Dirty team, worry about infecting with COVID-19, worry about family members infecting with COVID-19, worries about supplies, stigma. |
| Zaghini F, Fiorini J, Livigni L, Carrabs G, Sili A. 2021. | Italy  *n*=322 | COVID-19 hospitals/ February 19 to July 19, 2020 | A longitudinal mixed method | Nursing Questionnaire on Organizational Health (QISO) | - JS level: Post intervention Mean (SD)=3.05 (0.55), possible score is 1-4 - Impact of JS (+): - Impact of JS (-): NR - Factors affecting JS (+): Implementation of organization proactive management interventions - Factors affecting JS (-): NR |

**JS= Job Satisfaction, NR= Not Reported, SD= Standard deviation, +=Positive , and - = Negative**
